# Supplementary material for: Multi‐dataset identification of innovative feature genes and molecular mechanisms in keratoconus
Source: J Cell Mol Med. 2024 Sep 19;28(18):e70079. doi: 10.1111/jcmm.70079 (PMC11412914; doi:10.1111/jcmm.70079)
Supplement: Supplementary file 2 — Material S2. [file JCMM-28-e70079-s002.zip › 7.GSEA analysis/2.ATOH7/KEGG_PROTEIN_EXPORT.html]

Details for gene set KEGG\_PROTEIN\_EXPORT[GSEA]

|  || Dataset | ATOH7.group.cls#h\_versus\_l |
| Phenotype | group.cls#h\_versus\_l |
| Upregulated in class | l |
| GeneSet | KEGG\_PROTEIN\_EXPORT |
| Enrichment Score (ES) | -0.28501457 |
| Normalized Enrichment Score (NES) | -0.92689854 |
| Nominal p-value | 0.5772994 |
| FDR q-value | 1.0 |
| FWER p-Value | 1.0 |
Table: GSEA Results Summary

  

Fig 1: Enrichment plot: KEGG\_PROTEIN\_EXPORT      
 Profile of the Running ES Score & Positions of GeneSet Members on the Rank Ordered List

  

| SYMBOL | TITLE | RANK IN GENE LIST | RANK METRIC SCORE | RUNNING ES | CORE ENRICHMENT || 1 | OXA1L | na | 9892 | 0.083 | -0.1941 | Yes |
| 2 | SEC61A1 | na | 10595 | 0.072 | -0.1354 | Yes |
| 3 | HSPA5 | na | 10635 | 0.072 | -0.0582 | Yes |
| 4 | SPCS3 | na | 11796 | 0.055 | -0.0312 | Yes |
| 5 | SEC61G | na | 12046 | 0.052 | 0.0185 | Yes |
| 6 | SEC63 | na | 13650 | 0.034 | 0.0094 | Yes |
| 7 | SPCS2 | na | 13804 | 0.032 | 0.0405 | Yes |
| 8 | SRPRB | na | 14152 | 0.029 | 0.0623 | Yes |
| 9 | SEC11C | na | 14256 | 0.028 | 0.0898 | Yes |
| 10 | SPCS1 | na | 14788 | 0.023 | 0.0996 | Yes |
| 11 | SRP72 | na | 14990 | 0.021 | 0.1171 | Yes |
| 12 | SRP14 | na | 15790 | 0.014 | 0.1099 | Yes |
| 13 | SEC62 | na | 16440 | 0.009 | 0.1013 | Yes |
| 14 | SEC61B | na | 16535 | 0.008 | 0.1078 | Yes |
| 15 | SEC11A | na | 17636 | -0.001 | 0.0770 | Yes |
| 16 | SRP68 | na | 18027 | -0.004 | 0.0705 | Yes |
| 17 | IMMP2L | na | 19404 | -0.016 | 0.0486 | Yes |
| 18 | SRP9 | na | 19754 | -0.019 | 0.0598 | Yes |
| 19 | SRP19 | na | 20302 | -0.025 | 0.0712 | Yes |
| 20 | IMMP1L | na | 21125 | -0.033 | 0.0835 | Yes |
| 21 | SRP54 | na | 23581 | -0.063 | 0.0822 | Yes |
| 22 | SEC61A2 | na | 30208 | -0.218 | 0.1302 | Yes |
Table: GSEA details [plain text format]

  

Fig 2: KEGG\_PROTEIN\_EXPORT      
 Blue-Pink O' Gram in the Space of the Analyzed GeneSet

  

Fig 3: KEGG\_PROTEIN\_EXPORT: Random ES distribution      
 Gene set null distribution of ES for **KEGG\_PROTEIN\_EXPORT**
